# The Clinical Features and Long-Term Follow-Up of Vitamin B6-Responsive Infantile Spasms in a Chinese Cohort

**DOI:** 10.3389/fneur.2022.895978

**Published:** 2022-05-12

**Authors:** Xianru Jiao, Pan Gong, Yue Niu, Zhao Xu, Ye Wu, Yuehua Zhang, Zhixian Yang

**Affiliations:** Department of Pediatrics, Peking University First Hospital, Beijing, China

**Keywords:** vitamin B6, infantile spasms, West syndrome, epileptic spasms, hypsarrythmia

## Abstract

**Objective:**

To analyze the clinical features, treatment, and prognosis of patients with vitamin B6-responsive infantile spasms (IS).

**Methods:**

The clinical features, genetics, and follow-up data of 30 patients were collected and analyzed.

**Results:**

The age of epileptic spasms (ES) onset was from 3 months to 12 months. They all received high doses of vitamin B6 at different times after the onset of ES, ranging from 1 day to 5 months. ES were controlled within 11 days in 93% (28/30) patients, and as late as 1 month and 2 months in the other two patients. In the course of treatment, 28 patients were seizure-free all the time, and seizures of other two patients recurred due to withdrawal of vitamin B6. The available follow-up EEG results of 28 patients were normal in 26 cases, and 81% (21/26) had suppressed epileptic discharges within 6 months. Of the 26 cases with normal follow up EEG, 4 had developmental delay and 22 had normal development. The time for EEG to return to normal in 22 patients with normal development ranged from 14 days to 2 years (mean = 111.5 days; median = 52.5 days). The time for EEG to return to normal in the other 4 patients with development delay ranged from 4 months to 2 years (mean = 375 days; median = 330 days). To the last follow-up, seizures were controlled well in 29 surviving patients, and 21 patients were able to deactivate from all medications without seizures recurrence. Sixteen patients showed varying degrees of developmental delay after onset. After seizure control, the psychomotor development was delayed in 7 patients (one died) until the last follow-up. Genetic analysis did not show any meaningful results.

**Conclusion:**

An observation period of 1–2 weeks is essential to identify patients with vitamin B6-responsive IS. The treatment time could be extended according to the treatment response and EEG changes. It might take a longer time for EEG to return to normal and to stop taking drugs in patients with persistent or unimproved developmental delay. Neurodevelopmental outcomes and prognosis of vitamin B6-responsive IS were relatively favorable.

## Introduction

Infantile spasms (IS) classically present with a triad consisting of epileptic spasms (ES), a characteristic electroencephalogram (EEG) pattern known as hypsarrythmia or its variants, and neuro-regression (1). According to the report, partial patients diagnosed with IS responded to high-dose vitamin B6, and a series of similar cases have been reported (2–9). Following the availability of genetic studies, vitamin B6-dependent epilepsy, a heterogeneous group of disorders causing seizures that can be controlled or significantly improved by high doses of vitamin B6, including pyridoxine-dependent epilepsy (PDE), pyridox(am)ine-5′-phosphate oxidase *(PNPO)* deficiency and *PLPBP* deficiency, was formally diagnosed and treated (10–13). However, partial patients with IS do not have the genetic mutation characteristic of vitamin B6-dependent epilepsy, and they also dramatically respond to high-dose vitamin B6 therapy. IS controlled by vitamin B6 is termed as vitamin B6-responsive IS, and accounts for 10–30% of IS patients (7, 8).

Despite the long period of time since vitamin B6-responsive IS was discovered, there have been limited reports so far. Here, we describe the short- and long-term outcomes of 30 patients with vitamin B6-responsive IS, and 11 of these patients have been previously reported (14).

## Methods

### Ethics Statement

This study was approved by the Biomedical Research Ethical Committee of Peking University First Hospital. Written informed consent was obtained from the legal guardians (parents) of the patients.

### Patients

Thirty patients were diagnosed as vitamin B6-responsive IS in Peking University First Hospital between January 2012 and March 2021. The following criteria were established: (1) During the course of the disease, ES occurred and was the main seizure type; (2) Ictal EEG monitored isolated or series of ES; (3) Demonstration of hypsarrhythmia or atypical hypsarrhythmia (“atypical” or “modified” hypsarrhythmia was consist of asymmetric features, focal discharges, and semi-periodic burst-suppression); (4) Seizures could be controlled for at least 1 month while receiving vitamin B6 therapy alone or by the addition of vitamin B6 to previously ineffective antiseizure medications (ASMs) therapy; (5) The genetic test for vitamin B6-dependent epilepsy was negative, including *ALDH7A1, PLPBP*, and *PNPO*. For patients who could be managed and willing to cooperate, EEG monitoring was performed 1 week and 1 month after treatment to assess treatment response. Follow-up EEG was performed every 3–6 months until the result was normal. Brain magnetic resonance imaging (MRI), biochemical studies, plasma amino acids and urine organic acids test were performed in all patients. Neurodevelopmental assessment of these patients was evaluated through intelligence tests (Wechsler or Gesell intelligence scales). The intellectual disability (ID)/developmental delay (DD) severity was classified according to intelligence quotient (IQ) scores as mild (IQ of 55–70), moderate (IQ of 40–54), severe (IQ of 25–39), or profoundly severe (IQ of < 25).

### The Treatment of Vitamin B6

Since this study included a retrospective aspect, inconsistent treatments were covered. For patients who could be managed in our hospital, vitamin B6 was preferentially initiated in patients diagnosed as IS. For patients who have been treated with vitamin B6, but at an extreme low dose or for a short period of time (<7 days), vitamin B6 remained the primary treatment option.

Patients who met criteria 1–3 were given vitamin B6 at an initial dose of 10 mg/kg/day orally or 100–200 mg/day intravenously for at least seven days, while observing the efficacy, including seizure frequency and EEG. If the seizure was controlled after 1 week of vitamin B6 treatment, maintain the original dose of vitamin B6 treatment. If there was a significant reduction in the frequency of seizures within seven days or a significant improvement in EEG, vitamin B6 therapy was maintained and the observation period was extended to 2 weeks or longer, while the EEG was regularly reviewed. Other management protocols for IS could be added at the same time. If the seizure frequency did not change in the first week, then vitamin B6 would be discontinued and the next treatment was quickly moved on. At the same time, such patients were excluded from this study.

### Genetic Analysis

For 30 patients with vitamin B6-responsive IS, whole-exome sequencing (WES) was performed in 17 cases and gene panel test was performed in 13 cases. DNA extracted from peripheral blood from 17 patients and family members was analyzed using trio-based WES, which was performed at the local genetic institutes using next-generation sequencing. For other 13 patients who had undergone gene panel test, targeted sequencing was performed on genomic DNA samples from the proband and his/her parents for 535 epilepsy genes by MyGenostics Technology, Inc. (Beijing, China). Sequence variants were checked with population databases gnomAD (http://gnomad.broadinstitute.org/) and the Combined Annotation-Dependent Depletion (CADD, https://cadd.gs.washington.edu/) score, and functional impact of mutations was predicted by three different *in silico* tools, including Polyphen2, SIFT, and Mutation Taster. Variant pathogenicity was interpreted according to the American College of Medical Genetics (ACMG) guidelines (15).

## Results

A total of 30 patients were diagnosed with vitamin B6-responsive IS in our cohort. Clinical characteristics were summarized in Table 1. Genetic analysis of all patients was negative, either in the whole exon or the epilepsy-related genes panel including *ALDH7A1, PLPBP* and *PNPO*. We reanalyzed the available genetic data of nine patients, but the results were still negative.

**Table 1:** The clinical characteristics of patients with vitamin B6-responsive infantile spasms.

**Patient ID**	**Current age/gender**	**Age of spasm onset**	**The time from spasm onset to initiation of vitamin B6**	**The time from initiation of vitamin B6 to seizure free**	**ASMs of prior use of vitamin B6**	**ASMs added during vitamin B6 therapy/Time interval**	**Medication was used at the last follow-up**	**Seizure recurred**	**EEG at the last follow up**	**Duration to EEG return to normal**	**Brain MRI**	**Cognitive and Behavioral development**
Patient 1	7y9m/F	5m	3m	3d	TPM, VPA	/	/	No	Normal	3m	Normal	Normal
Patient 2	9y6m/M	8m	2m	1m	/	TPM/15d	/	No	Normal	1.5m	/	Normal
Patient 3	8y2m/M	7m	14d	7d	VPA	/	/	Yes	Normal	2y	Normal	Normal
Patient 4	6y9m/F	4m	13d	1d	/	/	/	No	Normal	1m	4m: Arachnoid cyst	Normal
Patient 5	15y/F	4m	2m	1d	VPA	/	/	No	Normal	10m	Normal	Mild delay
Patient 6	6y3m/F	3.5m	4m	3d	TPM, VPA, LEV, ACTH	/	/	No	Normal	1y	4m: Myelination delay	Severe delay
Patient 7	7y8m/F	5m	7d	4d	/	TPM/0d	/	No	Normal	4m	Normal	Normal
Patient 8	8y/M	5m	1m	5d	VPA, TPM	/	/	No	Normal	2.5m	Normal	Normal
Patient 9	7y8m/M	5m	15d	5d	/	TPM/2d	/	No	Normal	4m	Normal	Normal
Patient 10	9y6m/M	6m	1d	3d	/	/	/	No	Normal	11m	Normal	Normal
Patient 11	5y10m/F	4m	1m	3d	/	/	/	No	Normal	4m	/	Moderate delay
Patient 12	12y/M	12m	3m/5m	Inefficacy/7d	VPA, ACTH, TPM, LTG	/	Vitamin B6 (180 mg/day)	No	10y: Discharges in occipital and right anterior regions	n.a	1y3m: Extracranial space widen in frontal and temporal regions	Severe delay
Patient 13	5y/M	7m	1m	2d	/	/	/	No	Normal	1m	Normal	Normal
Patient 14	5y/M	4m	10d	2m	/	TPM/1.5m	/	No	Normal	6m	4m: Malacia in bilateral lateral ventricle posterior horn	Normal
Patient 15	3y9m/F	6m	2m	7d	/	TPM/0d	TPM (25 mg/day)	No	Normal	2m	Normal	Normal
Patient 16	4y10m/F	6m	1m	2d	/	/	/	No	Normal	6m	Normal	Normal
Patient 17	4y9m/F	6m	1m	1d	/	TPM/0d	/	No	Normal	1m	/	Normal
Patient 18	4y10m/M	4m	10d	4d	/	/	/	No	Normal	1m	Normal	Normal
Patient 19	4y6m/M	4m	1m	3d	/	TPM/0d	TPM (25 mg/day)	No	Normal	2y	5m: Extracranial space widen in frontal and temporal regions	Severe delay
Patient 20	Die at 1y3m/M	3m	2m	1d	LEV	/	n.a.	No	n.a	n.a	Normal	Severe delay
Patient 21	3y5m/M	5m	14d	1d	TPM	/	/	No	Normal	1.5m	Normal	Normal
Patient 22	5y4m/F	5m	10d	3d	/	LEV/1d	/	No	Normal	1m	7m: Extracranial space widen in right temporal region, right ventricles widened, Rathke cyst	Normal
Patient 23	1y9m/M	4m	8d	4d	/	TPM/2d	/	No	Normal	14d	Normal	Normal
Patient 24	3y/F	12m	1m	11d	/	TPM/7d	/	No	Normal	6m	1y3m: Abnormal white matter signals in bilateral parietal lobes	Normal
Patient 25	1y5m/M	5m	2d	1d	/	/	Vitamin B6 (100 mg/day)	No	Normal	2m	3m: Myelination delay	Normal
Patient 26	2y4m/F	3m	16d	5d	/	/	Vitamin B6 (60 mg/day)	No	n.a	n.a	Normal	Normal
Patient 27	1y2m/M	6m	7d	3d	/	/	Vitamin B6 (300 mg/day)	No	Normal	1m	Normal	Normal
Patient 28	2y/M	5.5m	8d	3d	/	TPM/3d	/	No	Normal	24d	Normal	Normal
Patient 29	8m/M	3m	3m	5d	/	TPM/5d	Vitamin B6 (40 mg/day)	No	Normal	1m	/	Normal
Patient 30	10y/M	6m	2m	3d	/	TPM/1d	TPM (50 mg/day)	Yes	3y: Discharges in right central and parietal regions, generalized fast waves	n.a	1y11m: Multiple encephalomalacia foci	Severe delay

*ASMs, antiseizure medications; EEG, electroencephalogram; MRI, magnetic resonance imaging; d, day; m, month; y, year; F, female; M, male; TPM, topiramate; VPA, valproic acid; LEV, levetiracetam; ACTH, adrenocorticotropic hormone; LTG, lamotrigine; n.a., not applicable*.

### Symptoms and Treatment

Of these 30 patients, 18 were male, and 12 were female. All patients had uneventful pre-and perinatal periods. The age of ES onset was from 3 months to 12 months (Table 1). Twenty-eight patients developed ES at onset and this was the only form of the disease in progress. Two other patients manifested as focal seizures initially at the age of 3 and 2 months, respectively (patient 12 and 20), followed by ES at 9 and 1 month later. Notably, patient 12 developed myoclonic seizures and atypical absence at 2 years of age.

All patients were treated with high-dose vitamin B6 at various times after ES onset, from 1 day to 5 months. Of 30 patients, 22 patients were not treated with any ASMs prior to vitamin B6, and nine of them received vitamin B6 monotherapy throughout the course of the disease. Four patients were treated with vitamin B6 and topiramate (TPM) simultaneously, and the episodes ceased at the 1, 3, 4, and 7 days, respectively after treatment. Of the other nine patients, the frequency of seizures was significantly reduced after treatment with vitamin B6, and then TPM or levetiracetam (LEV) was added within 1 to 45 days, after which the seizures disappeared. Additionally, eight patients were treated with multiple ASMs prior to vitamin B6, and only one patient (patient 12) had a transient response to ASMs treatment. Patient 12 developed focal seizures 3 months after birth and was controlled by oral valproic acid (VPA) treatment. Nine months later, the patient presented with ES, and VPA supplementation showed no effect. After 3 months, he was admitted to the hospital and given vitamin B6 intravenously for three consecutive days, but no effect was observed. Subsequently, ES was controlled after treatment with adrenocorticotropic hormone (ACTH). Then, this patient received VPA therapy and remained seizure-free for 1 year. At 2 years of age, ES recurred, and myoclonic seizures and atypical absence could be observed simultaneously. Multiple ASMs were ineffective. Five months later, he had no episodes after 7 days of intravenous vitamin B6. Since then, therapy with vitamin B6 and other ASMs was performed daily, and ASMs were gradually withdrawn after one and a half years without seizure recurred. By the time of the last follow-up (12 years of age), the patient had been seizure-free for nearly 10 years and vitamin B6 treatment was maintained (180 mg/day).

Of the 30 patients treated with vitamin B6, ES ceased completely within 24 h of life in 6 patients, 1–3 days in 11, 3–11 days in 11, and as late as 1 month and 2 months in two patients (patient 2 and 14), respectively. The two patients who required a relatively long time for seizure control underwent a long course of medication adjustment and observation. In case 2, the seizure was significantly reduced after vitamin B6 treatment, and was not improved after TPM [5.0 mg/(kg·day)] added at the age of 10 months. However, reducing the dose of vitamin B6 during treatment led to an increase in episodes and then restoring its dose to its original level. Finally, after the combination of vitamin B6 (60 mg/day) and TPM (25 mg/day), the seizures were completely controlled at the age of 11 months. Similarly, patient 14 developed ES episodes 4 months after birth and was treated orally with vitamin B6 10 days later. With vitamin B6 monotherapy, the frequency of ES was significantly reduced, but the patient still had a small number of isolated episodes occasionally. Subsequently, a small amount of TPM was added a month and a half later, and the seizures disappeared completely after 15 days.

### EEG and Brain MRI

The EEGs of all patients indicated hypsarrhythmia at onset. Follow-up EEG results were not available in 2 out of the 30 patients. One patient (patient 20) was due to early death, and the other (patient 26) did not undergo reexamination after high-dose vitamin B6 treatment. Of the 28 cases in which results were obtained, the corresponding EEG results of 26 patients were normal. In 21 out of 26 patients, the epileptic discharges were suppressed within 6 months and remained normal after the initiation of high-dose vitamin B6 therapy. Three patients' EEGs returned to normal within 10 months to 1 year. The EEG of the other two patients returned to normal after 2 years of vitamin B6 maintenance therapy. Of the 26 cases, 4 had developmental delay and 22 had normal development. The time for EEG to return to normal in 22 patients with normal development ranged from 14 days to 2 years (mean = 111.5 days; median = 52.5 days). The time for EEG to return to normal in the other 4 patients with development delay ranged from 4 months to 2 years (mean = 375 days; median = 330 days). Of the remaining two patients with abnormal EEG, the results of patient 12 indicated discharges in occipital and right anterior regions at the age of 10 years, and the seizure had been controlled for nearly 10 years, but the development is severely backward. The EEG results of patient 30 showed focal discharges in the right central and parietal regions, and generalized fast waves after the disappearance of hypsarrhythmia at the age of three, but no EEG review was available after several years of seizure control.

Brain MRI results of 26 patients were obtained, 17 of whom were normal. Nine patients showed different abnormalities at an earlier age, but were not reviewed at a later stage.

### Follow-Up and Neurodevelopment

During the follow-up (rang from 1 year to 10 years and 2 months), 28 patients were seizure-free all the time. The other two patients (3 and 30) had recurrent seizures due to an attempt to reduce vitamin B6 dose at 1 month and 8 months after treatment, respectively. The difference was that when treatment with vitamin B6 was resumed, the seizure of patient 3 was re-controlled, but the same effect was not seen in patient 30. The seizure of ES in patient 30 reoccurred after 2 months of vitamin B6 withdrawal. However, the addition of vitamin B6 failed to control the seizures, either orally or intravenously, and was eventually resolved by combination with TPM at the age of 2 years and 9 months and concurrent withdrawal of vitamin B6.

Up to the last follow-up, 29 patients survived, and one patient (case 20) with severe developmental delay died due to persistent fever and pneumonia 10 months later at the age of 1 year and 3 months. The age of the 29 surviving patients ranged from 8 months to 15 years. Seizures were controlled well in these 29 patients. Twenty-one patients were deactivated from all medications, including vitamin B6, at the last follow-up. Discontinuation of vitamin B6 was performed on a case-by-case basis. Twenty patients were desorbed with vitamin B6 between 8 months and two and a half years after seizure free. The other case (patient 23) showed a rapid decrease in vitamin B6 withdrawal, which was discontinued 2 months after seizure disappeared and two and a half months after EEG returned to normal. None of the 21 patients with vitamin B6 withdrawn had seizures recurrence. Five patients continued treatment with vitamin B6 monotherapy, and four of whom remained seizure free for approximately 2 months to 1 year prior to the last follow-up. Another patient (patient 12) maintained vitamin B6 treatment for nearly 10 years (180 mg/day) with abnormal EEG. For the remaining three patients (case 15, 19, and 30), vitamin B6 was withdrawn after a period of seizure control, and low-dose TPM monotherapy was maintained.

All patients showed normal neurodevelopment prior to epilepsy onset. After seizure onset, 16 patients showed varying degrees of developmental delay. Among them, nine patients improved gradually after the seizure was controlled, and had recovered to normal levels similar to those of their peers at the last follow-up. Up to the last follow up, the neurodevelopment was normal in 23, mild delay in 1, moderate delay in 1, and severe delay in 5 (one died).

## Discussion

Since Ohtahara first attempted to treat IS with high-dose vitamin B6, it had been recognized as the preferred treatment of choice in IS, especially in Japan (2, 16), which has been few changes in the last 20 years. In the past decade or so, treatment of IS patients with pyridoxine or pyridoxal phosphate has been reported intermittently (2–9). It's now generally accepted that vitamin B6 therapy should be tried first when IS was diagnosed, rather than other IS management protocols. Among IS patients, the proportion of patients who respond to high doses of vitamin B6 remains small. In this study, we reported 30 patients with vitamin B6-responsive IS, described and analyzed their clinical features, treatment processes, genetics, neurodevelopmental outcomes and follow-up data.

Ohtahara et al. (6) suggested that 100–200 mg/day or 20–30 mg/kg/day of vitamin B6 should be initiated in patients diagnosed as IS, and if efficacy is not apparent in the first week, it is better to increase the dose to 300–400 mg/day or 40–50 mg/kg/day, carefully observing seizure frequency and tolerability. In our cohort, all patients received an initial oral dose of vitamin B6 of 10 mg/kg/day or an intravenous dose of 100–200 mg/day. Obviously, we were getting the same treatment at a lower dose than reported internationally. Because the enrolled patients were treated in different hospitals before, the first treatment strategy was not uniform. In some patients, TPM and LEV were added at the beginning of vitamin B6 treatment or 1–7 days after treatment, which brings uncertainty to determine the efficacy of vitamin B6 treatment. Because the dosage of TPM or LEV was much lower than the minimum effective maintenance, we do not think it had strong enough effects to control the seizures in these patients. Nevertheless, we recommend that other drugs should not be added during the observation of the efficacy of vitamin B6 in IS patients, especially for the first 7 days, so as not to affect the judgment of effectiveness of vitamin B6.

In a previous observational study of vitamin B6-responsive IS, the effectiveness of vitamin B6 in controlling seizures was apparent within 1 week in 80% of patients, and by the 11th day in all responsive patients (8). Besides, Riikonen et al. showed that in responders, efficacy is observed within 1 to 2 weeks (9). Similarly, in our cohort, 93% of patients were under control within 11 days, and even 20% had their episodes completely resolved within 24 h of use. So, a 1–2-week observation period can identify more than 90% of patients with vitamin B6-responsive IS. Therefore, some scholars suggest that the efficacy of vitamin B6 is usually assessed after 1–2 weeks of use. If vitamin B6 does not work, move quickly to the next treatment. But in cases where vitamin B6 treatment has been effective, its effects are slow to show up in some patients. In our cohort, the spasms of two patients disappeared at 1 and 2 months, respectively, a little later than others. But during treatment, the frequency of seizures in both patients was significantly reduced, though not completely eliminated. Imai et al. reported on an IS patient who was treated with vitamin B6 for 30 days until the seizures disappeared (17). Therefore, in individual patients, the response to vitamin B6 is not rapid. At this time, it is necessary to extend the treatment cycle in time according to the curative effect and EEG changes.

However, in some cases, the efficacy of vitamin B6 therapy has not been properly evaluated. For example, the patient 12 in our cohort developed ES at 1 year of age and was treated with vitamin B6. However, as no effect was observed within 3 days, ACTH therapy was subsequently switched to. At 2 years and 5 months of age, recurrent seizures were controlled after seven days of treatment with vitamin B6. In addition, a case of IS reported by Imai et al. (17) experienced a similar situation. The patient was started the symptom at 9 months and received oral treatment of vitamin B6. However, because the EEG did not return to normal after a week, vitamin B6 was considered ineffective even without seizures. Treatment was subsequently switched to ACTH, and the seizures recurred at the age of one. Despite receiving a variety of conventional medications and a second ACTH treatment, her seizures were not suppressed. Vitamin B6 was treated again at 3 years and 6 months, and as a result she became seizure-free and hypsarrhythmia disappeared on EEG within a month. Then, Imai et al., suggested that a retrial of vitamin B6 at a later date could be worthwhile in cases of IS who had not responded previously and had relapsed after ACTH (17). However, the basic reason of the secondary treatment of vitamin B6 in these two patients was the short observation period of vitamin B6 treatment and misjudgment of efficacy. The most important factor in determining efficacy was whether the seizures controlled, not whether the EEG returned to normal, because the seizures usually stopped before the EEG returned to normal. Therefore, in the course of vitamin B6 treatment, it was important to pay close attention to changes in the frequency of seizures under sufficient observation time, and the change of EEG should be used as an auxiliary indicator.

Previous studies have shown that the EEG was important to monitor the course of treatment, in predicting relapse or in deciding whether to withdraw vitamin B6 therapy (8). Ohtahara et al. suggested that EEG examination at least once a week was recommended in the evaluation of the efficacy of vitamin B6 (8). Our previous study also suggested that EEG should be conducted weekly to assess the efficacy of initial treatment, and examined every 1–3 months to evaluate the therapeutic effect (14). This was also confirmed in our follow-up of 30 patients. Especially in patients whose EEG has not returned to normal for a long time, the EEG should be reviewed regularly to determine the progression of the disease. Regular EEG follow-up was known to predict the relapse of clinical seizures (8). Fortunately, in our cohort, worsening of EEG and recurrent seizures were not observed in all patients whose EEG returned to normal and vitamin B6 discontinuation.

The duration of vitamin B6 withdrawal depended on EEG changes. However, the timing of withdrawal of vitamin B6 and the outcome after discontinuation have not yet been clarified. The latest viewpoint was that two or more years being free of spikes on EEG might indicate a successful withdrawal of vitamin B6 (8). In our cohort, 21 patients of this series had successfully discontinued all medications after their EEG returned to normal, with no recurrence. In these 21 patients, epileptic discharges were suppressed within 2 years (range = 14 days to 2 years) after the initiation of high-dose vitamin B6 therapy, and 81% (17/21 cases) within 6 months. It could not be ruled out that the EEG of some patients might have returned to normal at an earlier time, but it has not been observed due to the absence of regular review. Then vitamin B6 was deactivated between 2.5 months and 2 years after EEG normalization. Considering most patients' EEG recovered within 6 months in our study, and that they discontinued vitamin B6 after another 1–2 years of maintenance therapy. For patients whose EEG returned to normal shortly after treatment, continuation of vitamin B6 for 1 to 2 years after EEG normalization is recommended. For individual patients with persistently abnormal EEG, the treatment period might be extended to 2 years or more. Cases of early withdrawal could be observed in previous studies, and their treatment cycle lasted only 6 weeks at an oral dose of vitamin B6 150 mg/day (9). Because of this, Riikonen et al. (9) suggested that if vitamin B6 was treated with a smaller dose than previously studied, it could be stopped after a relatively short treatment period in the absence of seizures or hypsarrhythmia EEG. All of our patients received lower doses of vitamin B6 than reported in the literature. However, spasms recurred due to vitamin B6 withdrawal were observed in two patients, after being controlled for 1 and 8 months, respectively, although the EEG was no longer hypsarrhythmia. Therefore, taking a relatively low dose of vitamin B6 still does not represent an early withdrawal.

IS was associated with poor neurodevelopmental outcome (1). A study of neurodevelopment in IS patients showed that regarding the cognition scale (Bayley-III developmental assessments), only 12% patients were within the normal range, and “severe delay” was found in 77% (18). In our cohort, when untreated or initially treated with vitamin B6, 53% patients showed varying degrees of developmental delay. At the last follow-up, 77% (23/30) patients were normal in neurodevelopment, which was prominently higher than that reported previously. This suggested that the neurodevelopmental outcomes and prognosis of vitamin B6-responsive IS were more favorable than those of other patients with IS. Besides, there were six patients in this group with severe developmental delay before treatment, and there was no significant difference in delayed treatment time. After long-term treatment, there was no significant change in developmental status in four cases, one was moderate, and one died. Therefore, for IS patients with severe developmental delay, even if the seizure was controlled by vitamin B6, the later development was difficult to improve to normal. In addition, four of seven patients with developmental delay had normal EEG at the last follow-up, two had abnormal EEG, and one died. Compared with other patients, EEG of these four patients took longer to return to normal, and vitamin B6 was stopped later or even failed to stop. Therefore, it might take a longer time for EEG to return to normal and to stop taking drugs in patients with persistent or unimproved developmental delay, even if there was no seizure after treatment.

### Limitations

Our study had several limitations. Due to the retrospective and prospective nature of this study, some patients received inconsistent treatment before the experimental treatment of vitamin B6, and even TPM and vitamin B6 combined treatment occurred. Lack of understanding of vitamin B6-responsive IS leads to differences in parental compliance and physician treatment guidelines, resulting in inconsistent intervals of EEG reexamination for most patients. During treatment, we neglected to monitor the side effects of vitamin B6. However, by describing the presentation of 30 vitamin B6-responsive IS, this study helped to made clinical recommendations on response observation period and treatment duration of vitamin B6, and formulate the standard of diagnosis and treatment for vitamin B6-responsive IS, which would be very helpful for clinical practitioners.

## Conclusion

In this study, we report 30 patients with vitamin B6-responsive IS. By describing and analyzing their clinical features and treatment processes, we reveal that an observation period of 1–2 weeks can identify more than 90% of patients with vitamin B6-responsive IS. At the same time, it is necessary to extend the treatment time in time according to the treatment response and EEG changes. For patients whose EEG returned to normal shortly after treatment, discontinuation of vitamin B6 1 to 2 years after EEG normalization is recommended. Neurodevelopmental outcomes and prognosis of vitamin B6-responsive IS were more favorable than those of other patients with IS. It might take a longer time for EEG to return to normal and to stop taking drugs in patients with persistent or unimproved developmental delay, even if there was no seizure after treatment.

## Data Availability Statement

The original contributions presented in the study are included in the article/supplementary material, further inquiries can be directed to the corresponding author.

## Ethics Statement

The studies involving human participants were reviewed and approved by Biomedical Research Ethical Committee of Peking University First Hospital. Written informed consent to participate in this study was provided by the participants' legal guardian/next of kin. Written informed consent was obtained from the minor(s)' legal guardian/next of kin for the publication of any potentially identifiable images or data included in this article.

## Author Contributions

ZY conceptualized and designed the study, coordinated the study overall, and revised the manuscript. XJ co-designed the study, drafted the initial manuscript, and revised the manuscript. PG, YN, ZX, YW, and YZ helped to collect and summarize data and revised the manuscript. All authors approve of the final revision of the article.

## Funding

This work was supported by National Nature Science Foundation of China (82171436), Beijing Natural Science Foundation (7202210), and Capital's Funds for Health Improvement and Research (2020-2-4077).

## Conflict of Interest

The authors declare that the research was conducted in the absence of any commercial or financial relationships that could be construed as a potential conflict of interest.

## Publisher's Note

All claims expressed in this article are solely those of the authors and do not necessarily represent those of their affiliated organizations, or those of the publisher, the editors and the reviewers. Any product that may be evaluated in this article, or claim that may be made by its manufacturer, is not guaranteed or endorsed by the publisher.
